# The differential role of cortical protein synthesis in taste memory formation and persistence

**DOI:** 10.1038/npjscilearn.2016.1

**Published:** 2016-05-11

**Authors:** David Levitan, Shunit Gal-Ben-Ari, Christopher Heise, Tali Rosenberg, Alina Elkobi, Sharon Inberg, Carlo Sala, Kobi Rosenblum

**Affiliations:** 1 Sagol Department of Neurobiology, Center for Gene Manipulation in the Brain, University of Haifa, Mt Carmel, Haifa, Israel; 2 Consiglio Nazionale delle Ricerche Neuroscience Institute and Department of Biotechnology and Translational Medicine, University of Milano, Milan, Italy

## Abstract

The current dogma suggests that the formation of long-term memory (LTM) is dependent on protein synthesis but persistence of the memory trace is not. However, many of the studies examining the effect of protein synthesis inhibitors (PSIs) on LTM persistence were performed in the hippocampus, which is known to have a time-dependent role in memory storage, rather than the cortex, which is considered to be the main structure to store long-term memories. Here we studied the effect of PSIs on LTM formation and persistence in male Wistar Hola (*n*⩾5) rats by infusing the protein synthesis inhibitor, anisomycin (100 μg, 1 μl), into the gustatory cortex (GC) during LTM formation and persistence in conditioned taste aversion (CTA). We found that local anisomycin infusion to the GC before memory acquisition impaired LTM formation (*P*=8.9E−5), but had no effect on LTM persistence when infused 3 days post acquisition (*P*=0.94). However, when we extended the time interval between treatment with anisomycin and testing from 3 days to 14 days, LTM persistence was enhanced (*P*=0.01). The enhancement was on the background of stable and non-declining memory, and was not recapitulated by another amnesic agent, APV (10 μg, 1 μl), an *N*-methyl-D-aspartate receptor antagonist (*P*=0.54). In conclusion, CTA LTM remains sensitive to the action of PSIs in the GC even 3 days following memory acquisition. This sensitivity is differentially expressed between the formation and persistence of LTM, suggesting that increased cortical protein synthesis promotes LTM formation, whereas decreased protein synthesis promotes LTM persistence.

## Introduction

Memory is composed of phases characterized by their sensitivity to molecular and behavioral perturbations, such as protein synthesis inhibitors (PSIs). Long-term memory (LTM) formation is dependent on transcription and translation processes taking place around the time of memory acquisition.^1–4^ However, the involvement of protein synthesis in LTM persistence is poorly understood, as a memory trace increases its stability to PSIs as the time interval between memory acquisition and PSI infusion increases.^5–8^

Interestingly, recent studies challenge this view, showing that PSIs can affect LTM persistence up to 24 h post acquisition, if they are tested with a delay of few days.^5–8^ Injection of anisomycin, a classic and widely used PSI, to the hippocampus during an inhibitory avoidance learning paradigm up to 24 h post acquisition resulted in LTM impairment evident only 7 days but not 2–3 days later.^6,8^ This interesting study has opened the door for measuring new variables: different phases of sensitivity to PSIs and different time intervals between acquisition and the time point when memory is measured.

This study used PSI injections to the hippocampus, which is known to be involved in an additional process of system memory consolidation.^9,10^ This suggests that LTM can be sensitive to the action of PSIs even >24 h post-acquisition, if administered to cortical regions, which are considered to store LTM traces persistently, at least partially.^11^

The gustatory cortex (GC), which resides in the anterior insular cortex, is crucial for learning the association between a taste and potential visceral discomfort or pain. This form of associative learning is termed conditioned taste aversion (CTA), and is measured in the lab when a normally appetitive taste (e.g., saccharin) becomes aversive after being paired with gastric distress (e.g., using lithium chloride). Importantly, the formation of LTM and its long-term retrieval during CTA is dependent on the function of the GC.^12–14^ Moreover, intact protein synthesis in the GC has an indispensable part in this form of learning.^15–17^

In the present study, we studied the effect of GC cortical protein synthesis inhibition on CTA LTM formation and persistence by stereotactically infusing the protein synthesis inhibitor, anisomycin, into the GC during the different stages of LTM.

## Results

We used CTA, a behavioral paradigm in which a normally appetitive taste (e.g., saccharin) becomes aversive after being paired with gastric distress (e.g., induced by lithium chloride). The formation of LTM and its long-term retrieval in this paradigm is dependent on the function of the GC,^12–14^ with protein synthesis playing an indispensable part.^15–18^

**Figure 1:**
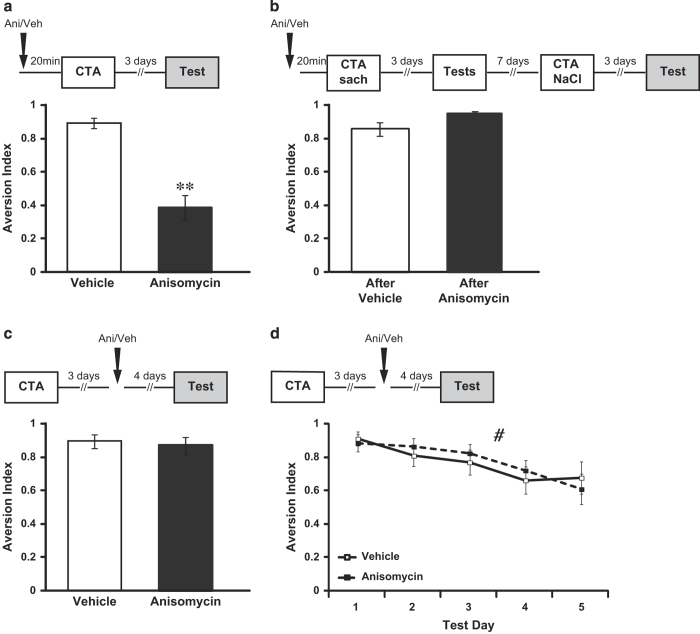
Stereotactic infusion of anisomycin into the gustatory cortex (GC) before acquisition impairs long-term memory, whereas infusing it 3 days later has no effect. (**a**) Anisomycin (*n*=6) infusion to the GC 20 min before conditioned taste aversion (CTA) results in a lower aversion index (AI) than in vehicle-infused (*n*=6) rats. **P=8.9E−5. (**b**) One week following the completion of CTA for saccharin both groups (*n*=6) were subjected to CTA for a different and novel taste (0.3% NaCl), which resulted in higher aversion toward NaCl with no significant difference between them. (**c**) Anisomycin (*n*=9) or vehicle (*n*=11) were infused to the gustatory cortex 3 days following CTA acquisition. Both groups showed high aversion with no significant difference between them when tested 4 days later. (**d**) On continued testing for four more consecutive days both groups acquired extinction to saccharin (which is expressed as a significant difference between day1 and day5) with no significant difference between them. ^#^ *P*=0.845.

We infused the protein synthesis inhibitor, anisomycin, to the GC to temporally block protein synthesis during the different stages of CTA LTM. Previously, we have found that a single infusion of the drug to the GC inhibits protein synthesis significantly for at least 4 h.^15^ To test the effect of anisomycin on LTM formation, we infused it or vehicle (saline) to the GC 20 min before memory acquisition (Figure 1a). When tested 3 days later, animals receiving anisomycin showed significantly lower aversion toward saccharin than animals receiving vehicle infusion (*n*=6 per group, *t*_10_=6.3, *P*=8.9E−5, *t* test).

To test whether anisomycin affects memory or taste perception, we retrained the same animals in the CTA paradigm for a different taste (NaCl) without anisomycin treatment. Both groups demonstrated high aversion toward NaCl with no significant difference between them (Figure 1b: *n*=6 per group, *t*_10_=−2; *P*=0.071, *t*-test). This indicates that, indeed, anisomycin affected LTM specifically, and that its infusion did not inflict permanent damage on the GC and taste perception.

Next, we tested the effect of anisomycin on LTM persistence by infusing it or vehicle to the GC 3 days following memory acquisition (Figure 1c). When tested 4 days later, both groups showed high aversion toward saccharin with no significant difference between groups (*n*=9–11, Mann–Whitney: *Z*=−0.113; *P*=0.94). We continued testing once daily for four consecutive days to assess the ability of the animals to form extinction of the aversive response to saccharin (Figure 1d). Both groups showed extinction, expressed as a significant difference between day 1 and day 5 in each group (Mann−Whitney: Anisomycin: *n*=9; Z=−4.01, *P*=0.0001; Vehicle: *n*=11; Z=−3.72, *P*=0.0001). Moreover, there was no significant difference between the groups (analysis of variance (ANOVA): F_1,18_=0.39; *P*=0.845).

Studies that tested the influence of protein synthesis inhibitors thus far assumed that the inhibitors can only impair memory, but not enhance it. This can be inferred from the use of protocols, which resulted in strong memory, producing a ceiling effect and excluding the possibility of revealing memory enhancement.^15–18^

**Figure 2:**
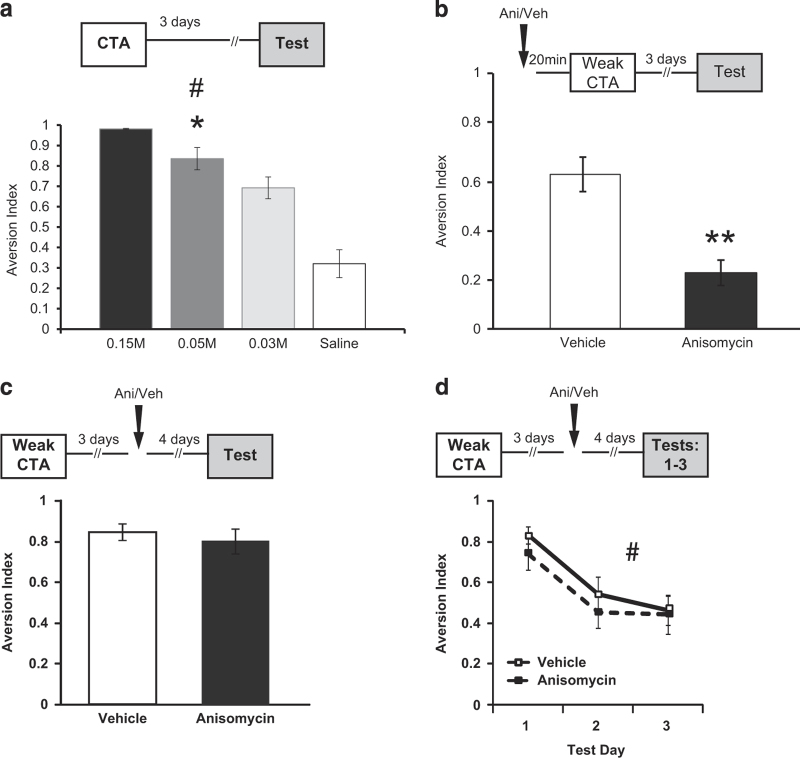
Injection of anisomycin into the gustatory cortex (GC) before weak conditioned taste aversion (CTA) protocol impairs long-term memory, whereas infusing it 3 days later has no effect. (**a**) Weak CTA protocol: reducing lithium chloride (LiCl) concentration from 0.15 mol/l to 0.05 mol/l or 0.03 mol/l during CTA training results in lower aversion index (AI) when tested 3 days later, thus allowing detection of both enhancement and impairment of memory. 0.15 mol/l, *n*=15; 0.05 mol/l, *n*=5; 0.03 mol/l, *n*=9. Main effect of group, analysis of variance (ANOVA), ^#^ *P*=0.0001. *P=0.026 between 0.15 mol/l and 0.05 mol/l. *P*=0.047 between 0.05 mol/l and 0.03 mol/l. (**b**) Weak CTA long-term memory (LTM) formation is sensitive to protein synthesis inhibition. Animals were trained with 0.05 mol/l of LiCl (weak CTA protocol) 20 min after anisomycin or vehicle injection to the GC. Testing the animals 3 days later resulted in memory impairment (Figure 4e: *n*=8 per group, *t*_15_=12.6, **P=0.0001, *t* test), demonstrating that the sensitivity of LTM to protein synthesis inhibition before memory acquisition applies for weak CTA protocol as well. (**c**) Anisomycin (*n*=9) or vehicle (*n*=13) were infused to the gustatory cortex 3 days following weak CTA acquisition. Both groups showed lower aversion than standard CTA (see text) with no significant difference between them when tested 4 days later. (**d**) On continued testing for two more consecutive days both groups acquired extinction for saccharin aversiveness (which is expressed as a significant difference between day1 and day3) with no significant difference between them ^#^ *P*=0.474.

To test for a possibility of memory enhancement, we used modified, weak protocols for CTA, in which LiCl concentration was reduced from 0.15 mol/l to 0.05 mol/l or 0.03 mol/l (Figure 2a). Using these lower concentrations of LiCl resulted in lower aversion index (AI) compared with the higher dose of LiCl, allowing detection of both enhancement and impairment of memory (0.15 mol/l, *n*=15; 0.05 mol/l, *n*=5; 0.03 mol/l, *n*=9. Main effect of group, ANOVA, *P*=0.0001. *P*=0.026 between 0.15 and 0.05 mol/l). It is important to note that we found variation in the AI scores across different batches using the weak CTA protocol. This variation can also be inferred from previous CTA studies from our lab^14,19^ and others.^20^ For this reason we always compared groups from the same batch, and used batches with an average AI scores <0.83, which was lower enough to reveal memory enhancement (such as between 0.15 and 0.05 mol/l CTA in Figure 2a).

Training animals with a weak CTA protocol (0.05 mol/l LiCl) 20 min after anisomycin injection to the GC resulted in memory impairment when tested 3 days later (Figure 2b: *n*=8 per group, *t*_15_=12.6, *P*=0.0001, *t* test), demonstrating that the sensitivity of LTM formation to protein synthesis inhibition before memory acquisition occurs as a result of weak CTA protocol as well as the standard CTA protocol.

To test whether anisomycin can enhance LTM persistence, we infused it or vehicle to the GC 3 days following a weak CTA protocol, wherein 0.05 mol/l LiCl was used (Figure 2c). When tested 4 days later, animals receiving anisomycin showed no significant difference compared with animals receiving vehicle infusion (*n*=9–11; *t*_20_=−0.585; *P*=0.56, *t* test). We continued testing once daily for two consecutive days to assess CTA extinction (Figure 2d). Both groups showed extinction, expressed as a significant difference between day1 and day3 (anisomycin: *n*=9, *t*_8_=5.26, *P*=0.001; vehicle: *n*=1, *t*_12_=4.95, *P*=0.0001, *t* test). Moreover, we found no significant difference between the groups (ANOVA: F_1,20_=0.53; *P*=0.474). Thus, so far, we have repeated the major findings of past experiments regarding the influence of protein synthesis inhibitors on LTM formation and persistence.^2,4,21–23^ We showed that CTA formation is sensitive to the protein synthesis inhibitor, anisomycin, but that CTA persistence is not.

**Figure 3:**
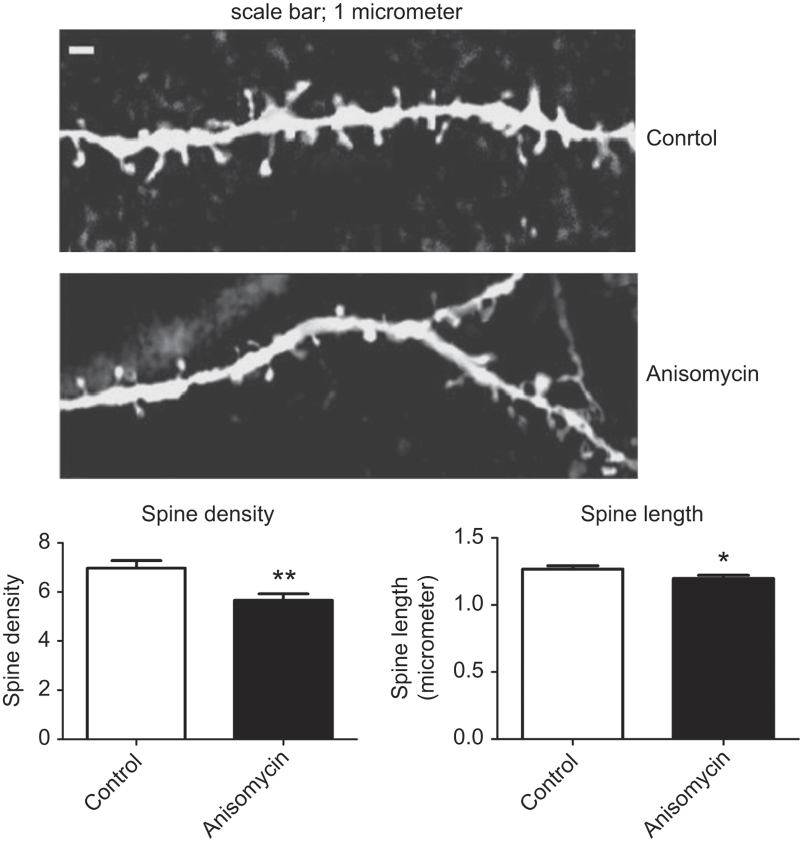
Anisomycin infusion to the gustatory cortex reduces spine density and length. Anisomycin or vehicle was infused to the rat gustatory cortex and brains were subjected to Golgi staining 6 days later. Anisomycin-infused animals showed significant reduction both in spine density (**P=0.0017, *t*=3.299, degrees of freedom (d.f.)=57) and spine length (*P=0.0336, *t*=2.141, d.f.=56). The analysis is based on *N*=28–32, where *N*=analyzed neuron, taken from four animals in each condition.

The increasing resistance of LTM to protein synthesis inhibitors is rather surprising, as recent studies have shown that protein synthesis inhibitors can have a profound effect on synaptic morphology,^11,24–27^ which is thought to be the substrate of memory. To test whether the effect on morphology could apply to the GC as well, we infused anisomycin to the GC of naïve animals and analyzed spine morphology 6 days later. Anisomycin infusion resulted in reduction of spine density and length as compared to vehicle (Figure 3. density: *P*=0.0017, *t*=3.299, degrees of freedom (d.f.)=57; length: *P*=0.0336, *t*=2.141, d.f.=56, *n*=28–32 neurons from four animals in each condition; *t* test with Welch correction). These results are in accordance with a previous report, which found the same trend 4 days following anisomycin infusion to the motor cortex.^26^

**Figure 4:**
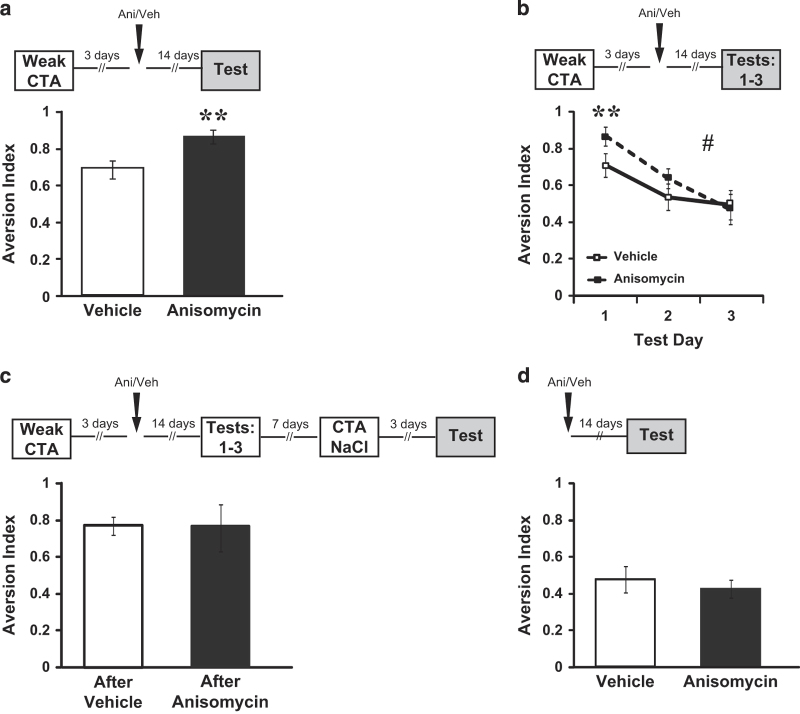
Increasing the time interval between anisomycin infusion to memory testing results in long-term memory enhancement. (**a**) Anisomycin (*n*=14) or vehicle (*n*=16) was infused to the rat gustatory cortex 3 days following weak conditioned taste aversion (CTA) acquisition. When tested 14 days later, animals receiving anisomycin showed significantly higher aversion toward saccharin than animals receiving saline vehicle infusion. **P=0.01. (**b**) On continued testing for two additional consecutive days both groups acquired extinction of aversion to saccharin (**P=0.001 for anisomycin, **P=0.005 for vehicle) with no significant difference between them. ^#^ *P*=0.182. (**c**) One week following the completion of CTA for saccharin both groups (only last batch of animals. *n*=5) were subjected to CTA for a different and novel taste (0.3% NaCl) which resulted in aversion toward NaCl with no significant difference between the groups. (**d**) Aversion to saccharin in naive rats is not influenced when tested 14 days following infusion of anisomycin to the gustatory cortex (*n*=15) as assessed by comparison to vehicle-infused rats (*n*=18).

In previous studies, the time interval between infusion of PSIs to memory testing was 1–4 days, poorly addressing the possibility of a long-term effect on the persistence of memory. Interestingly, delayed testing of the memory trace can unmask the effect PSI’s on LTM persistence.^6–8^ Therefore, we tested this possibility. We infused anisomycin or vehicle to the GC 3 days following CTA acquisition. Surprisingly, when tested 14 days later, animals receiving anisomycin showed significantly higher aversion towards saccharin than animals receiving vehicle infusion (Figure 4a: *n*=14–16, Mann–Whitney: Z=−2.534; *P*=0.01). These results suggest that protein synthesis inhibition enhances LTM evident 2 weeks but not 4 days after PS infusion.

We continued testing for two additional consecutive days to measure CTA extinction. Both groups showed extinction, expressed as a significant difference between day1 and day3 in each group (Figure 4b: *n*=14–26, Mann–Whitney: anisomycin: *Z*=−3.23, *P*=0.001; vehicle: *Z*=−2.79, *P*=0.005). Moreover, we found no significant difference between the groups over the three test days (ANOVA: F_1,28_=1.82; *P*=0.182), indicating there is no significant difference in the extinction rate between the anisomycin- and vehicle-infused groups.

One week after the test we retrained the animals for a different and novel taste (NaCl). Testing the animals 3 days later revealed that both groups showed high aversion with no significant difference between them (Figure 4c: *n*=5, Mann–Whitney: *Z*=−0.523; *P*=0.69). The ability of the anisomycin-infused animals to form extinction for one taste, formerly treated as aversive and an aversive memory for a different taste, both of which are dependent on the integrity of the GC,^17^ indicates no permanent damage to this brain structure and intact behavioral plasticity following anisomycin infusion.

To test whether anisomycin-induced memory enhancement results from its interaction with memory or from creating a general state of aversion, we tested the effect of anisomycin in naive animals. We infused anisomycin or saline to the GC (Figure 4d), and tested the naive animals for aversion to saccharin as in our previous memory tests. There was no significant difference between the groups (*n*=15–18, Mann–Whitney: *Z*=−0.353; *P*=0.8). Moreover, in a similar experiment we tested naive animals for aversion to a bitter taste, quinine. We found no significant difference between the groups (*t* test: *t*_10_=0.518, *P*=0.616; anisomycin *n*=5, aversion index (AI)=97%; vehicle *n*=7, AI=97%). The ability of the anisomycin-infused animals to respond normally both to aversive and to appetitive tastes indicates that anisomycin has no effect on taste recognition 14 days following the infusion. Furthermore, it indicates that anisomycin interacts with an already established memory to enhance it.

**Figure 5:**
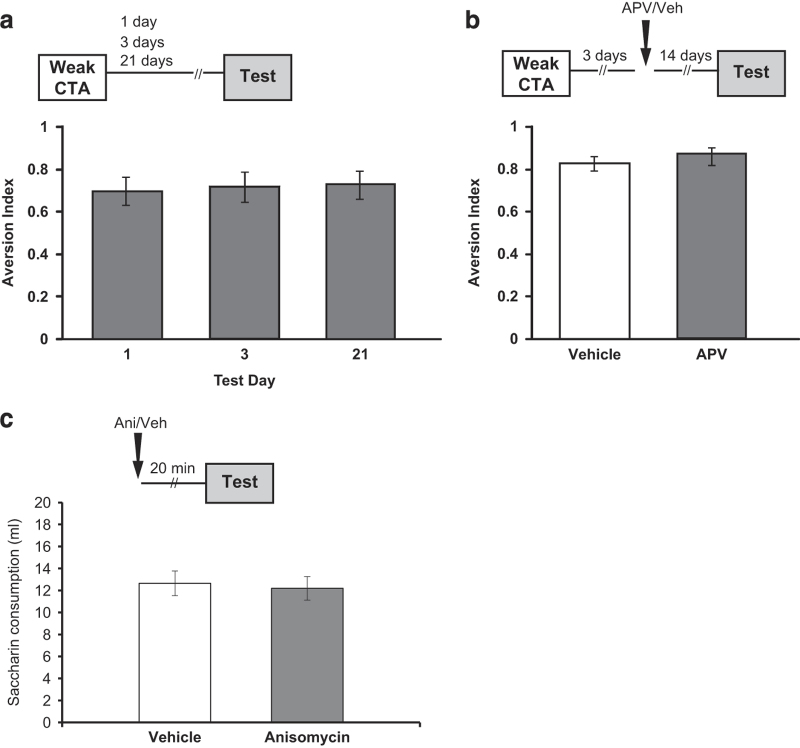
Memory enhancement is on the background of a stable and non-declining memory and is not susceptible to other interruptions to memory consolidation. (**a**) There is no significant difference between groups of animals that were tested 1, 3, and 21 days following weak conditioned taste aversion (CTA) (1 day *n*=15, 3 days *n*=11, 21 days *n*=14; Kruskal–Wallis test: *χ*^2^=0.016; *P*=0.99). (**b**) APV (*n*=11) or vehicle (*n*=12) was infused to the gustatory cortex (GC) 3 days following CTA acquisition. Both groups showed aversion with no significant difference between them when tested 4 days later (*t* test: *t*_21_=0.63, *P*=0.54). (**c**) Anisomycin injection to the GC has no effect on 0.1% saccharin consumption. Animals were injected with anisomycin (*n*=6) or vehicle (*n*=8) to the GC 20 min before 0.1% saccharin consumption. There was no significant difference between the groups (*P*=0.78).

The enhancement of memory could be on the background of a stable or declining memory, where each possibility may result in a different interpretation. To test the different possibilities, we compared memory for weak CTA over time (Figure 5a). We found no significant difference between groups of animals that were tested 1, 3, and 21 days following weak CTA (*n*=15, Kruskal–Wallis test: *χ*^2^=0.016; *P*=0.99). Thus, the enhancement of LTM following anisomycin infusion to the GC three days after acquisition is on the background of a stable and non-declining memory.

The above results suggest that different mechanisms underlie LTM formation and persistence, as they are differentially influenced by anisomycin. Next we wanted to further test whether LTM persistence could be differentially affected by another LTM formation blocker, APV (D,L-2-amino-5-phosphnovaleric acid), an *N*-methyl-D-aspartate (NMDA) receptor channel antagonist. Importantly, the infusion of APV to the GC before CTA acquisition results in LTM impairment.^17^ We tested whether APV infusion to the GC could affect CTA memory, if infused three days following acquisition and tested 14 days later. As shown in Figure 5b, APV-infused animals showed no significant difference from vehicle-infused animals (*n*=11–12, *t* test: *t*_21_=0.63, *P*=0.54). Thus, persistence of CTA memory is not dependent on NMDA receptor activity, further suggesting a differential mechanism for LTM formation and persistence.

## Discussion

It is thought that the formation of LTM in different species and brain structures is dependent on protein synthesis, whereas its long-term persistence is not. However, here we show evidence for the continuous sensitivity of LTM to the action of protein synthesis inhibitors (PSIs) in the GC in the CTA learning paradigm. This sensitivity to PSIs lasts from the time of memory acquisition to at least 3 days later, encompassing LTM formation and persistence, Importantly, although the formation of LTM was inhibited, its persistence was enhanced, suggesting that an increase in protein synthesis promotes LTM formation, whereas reduced protein synthesis promotes LTM persistence.

Here we studied the long-term effect of PSIs on LTM by stereotactically infusing anisomycin, a protein synthesis inhibitor, to the GC just before or 3 days after CTA acquisition. In agreement with other CTA studies,^15,17^ as well as in other paradigms,^7,28,29^ there was decreased sensitivity of the memory trace to anisomycin with time. Alhough anisomycin injected to the GC around the time of memory acquisition decreased the strength of LTM formation, infusion of anisomycin 3 days after memory acquisition had no effect. As in previous experiments, the time interval between PSI administration and testing was 1–4 days.^7,15–18,28,29^ Surprisingly, when we extended this interval from 4 to 14 days, LTM persistence was enhanced, indicating that: (1) intact protein synthesis in the GC has an important role in LTM persistence in the CTA paradigm; (2) reduction in protein synthesis, rather than increase, promotes LTM persistence.

Memory may have been enhanced due to non-specific mechanisms affecting taste behavior instead of memory. We find this possibility unlikely since: (1) Taste recognition in naïve animals 14 days following anisomycin infusion was intact. (2) Animals in which memory was enhanced could extinguish the aversive trace and then could form a novel aversive memory, implying intact behavioral plasticity. Altogether these results suggest that anisomycin infused 3 days following acquisition specifically enhances LTM persistence without affecting and extinction or taste recognition.

The enhancement of memory could be on the background of a stable or declining memory, where each possibility may result in a different interpretation. The first possibility implying for effect on memory maintenance and the later on memory forgetting. Here we found that, with the protocol we have used to induce weak CTA, memory has not declined over the course of 3 weeks. Thus, indicating that it is the persistence of memory and not its forgetting that was affected.

Recently, it was reported that in addition to protein synthesis inhibition, anisomycin can block neuronal activity,^30^ raising the possibility that it affected memory by silencing activity in the GC rather than protein synthesis. This possibility is highly unlikely in this study for the following reasons: (1) We used anisomycin, which inhibits >80% of GC protein synthesis for at least 4 h *in vivo*.^15^ (2) Anisomycin injection to the GC reduced spine density and length (Figure 3), a phenotype associated with protein synthesis inhibition in previous studies.^24–27^ (3) Anisomycin had no effect on the drinking volume during memory acquisition (Figure 5c) and CTA retrieval,^15^ both of which should be attenuated by GC silencing.^12,31^

Little is known about the mechanisms of LTM persistence and in particular, the role of protein synthesis in this processes. Recently, it was demonstrated that LTM can be sensitive to the action of PSIs in a relatively remote time point (days after memory acquisition) than had been demonstrated before (up to few hours after memory acquisition), thus affecting LTM persistence and not only formation.^6,8^ Our results are in line with these studies, demonstrating that LTM persistence is sensitive to GC injection of anisomycin. However, in contrast to former studies, here we describe memory facilitation rather than inhibition, a previously unappreciated role for protein synthesis in modulating LTM. The fact that protein synthesis inhibition has a differential effect on LTM formation and persistence indicate that protein synthesis has a dynamic role during the different stages of LTM and adds to the accumulated data regarding differential molecular mechanisms for LTM formation and persistence.^20,32,33^

Interestingly, we found that the infusion of another amnestic agent, APV, an NMDA antagonist, into the GC does not recapitulate the action of anisomycin on LTM persistence. APV and anisomycin injection to the GC before CTA impaired CTA. However, only the injection of anisomycin 3 days following memory acquisition enhanced LTM persistence tested 14 days later. APV^4,34^ and anisomycin^15^ influence LTM formation through translation regulation. However, although the former acts indirectly, blocking signal transduction from the plasma membrane to the ribosome, the latter acts directly on the translation elongation processes. This suggests that protein synthesis is modulated through NMDA receptor activity during LTM formation but not persistence, further suggesting a differential mechanism for LTM formation and persistence.

Protein synthesis in the GC and the amygdala is a key for CTA LTM formation.^35^ Neurons from both of this structures form a reciprocal circuit, which undergoes long-term plasticity *in vivo* and that has indispensable part in CTA memory.^36^ This might suggest that protein synthesis in the amygdala has a role in LTM persistence along with LTM formation. Other brain regions integral to the taste system, such as the prefrontal-cortex^37^, might also regulate protein synthesis during LTM persistence, and in fact may have opposite effect than the GC in regulating CTA. Thus, it would be important to test whether protein synthesis in amygdala and the prefrontal-cortex neurons could have the same or opposite role as the GC during memory maintenance of CTA.

Here we demonstrate that GC protein synthesis inhibition had effect on CTA at later time point (3 days after memory acquisition) than evident when inhibiting protein synthesis in the hippocampus during spatial learning^6,8^ (up to 24–48 h after memory acquisition). This difference in the sensitivity between the cortex and the hippocampus is consistent with the notation that the hippocampus has a limited role in spatial or fear conditioning learning paradigms, as opposed to the cortex that has a permanent role in memory storage.^9–11^ The need for intact protein synthesis in LTM formation is universal^1–5,21–23^ (general need of translation process around the time memory acquisition), and correct also for the function of the GC in CTA consolidation.^15–18^ The sensitivity of CTA LTM persistence to protein synthesis inhibition in the GC may indicate a more general role for protein synthesis during LTM persistence, namely, enhancement of LTM persistence when injected 3 days after memory acquisition to other cortical regions and tested in other learning paradigms. However, additional experiments are needed to reveal the extent of generalization to other learning paradigms and other brain regions. It would be important to test whether protein synthesis inhibition in other brain structures that has a role in memory formation in other learning paradigms, such as the mPFC during inhibitory avoidance^37^ and the amygdala during fear conditioning^38^ could enhance memory maintenance.

Collectively, this study and those of Bekinschtein *et al.*^6^ and Bambah-Mukku *et al.*^8^ point to a novel concept of LTM persistence. The fact that LTM persistence is initially impaired by protein synthesis inhibition^6,8^ (1 day following memory acquisition) and is subsequently enhanced (3 days following memory acquisition—this study) is interesting and important. It suggests that LTM persistence has at least two different time phases, each with a different underlying molecular mechanism. In the future, it would be important to investigate the temporal change of LTM persistence sensitivity to protein synthesis inhibition and determine the molecular mechanisms underlying the different phases of LTM persistence.

In conclusion, the results of this study indicate that LTM, through LTM formation and persistence, remains sensitive to protein synthesis inhibition in the cortex from the time of memory acquisition to at least 3 days later, pointing to a hitherto unappreciated role for protein synthesis in modulating LTM persistence. The sensitivity is differentially expressed between LTM formation and persistence, suggesting that an increase in protein synthesis promotes LTM formation, whereas reduced protein synthesis promotes LTM persistence. Further experiments are necessary to determine the molecular mechanisms underlying this new and uncharacterized stage of LTM persistence.

## Materials and methods

### Animals

Male Wistar Hola rats (6–8 weeks of age; 250–300 g) were housed individually, on a 12-h light/dark cycle, *ad libitum*. Animals were handled according to approved protocols and animal welfare regulations of the University of Haifa Institutional Ethics Committee.

Saline (0.9% NaCl) was used as vehicle. Anisomycin (Sigma, Rehovot, Israel) was dissolved in equimolar HCl, adjusted to pH 7.2 and brought to a final concentration of 100 μg/μl in saline. APV was dissolved in saline and adjusted to pH 7.2 and brought to a final concentration of 10 μg/μl.

### Behavioral paradigm

CTA experiments were performed as described previously.^15,16^ Briefly, animals underwent 3 days of water restriction training, receiving 20 min access to 20 ml of water in two 10 ml pipettes daily. On the fourth day the animals were given 20 min 0.1% saccharin and 40 min after the termination of drinking received an intraperitoneal injection of malaise-inducing agent LiCl (0.15 mol/l 2% of body weight unless indicated otherwise). Three days before testing the water restriction training was resumed. On the day of testing, rats received a 20 min test choice between 20 ml 0.1% saccharin and 20 ml water. Memory was expressed as an aversion index to saccharin, calculated as the amount of water consumed divided by total fluid consumption ((water volume)/(water+saccharin)).

### Surgery and microinfusion

Rats were anesthetized with equithesin (0.45 ml per 100 g; 2.12% (w/v) MgSO_4_, 10% (v/v) ethanol, 39.1% (v/v) sodium pentobarbital and 4.2 (w/v) chloral hydrate), restrained in a stereotactic apparatus (Stoelting, Wood Dale, IL, USA) and were implanted bilaterally in the gustatory cortex with 23-gauge stainless-steel guide cannulas (coordinates, relative to bregma: AP=+1.2 mm, *L*±5.5 mm, *H*=5.5 mm). After surgery, the animals were allowed 1 week to recover before the experimental manipulation.

For microinfusion, a 28-gauge injection cannula was carefully inserted 1 mm beyond the tip of the guide cannula, and was connected via PE20 tubing to a Hamilton microsyringe driven by a CMA/100 microinjection pump (Carnegie Medicine, Stockholm, Sweden), that injected at 1 μl/min. Following injection, the injection cannula was left in place for an additional 1 min before withdrawal, to minimize leakage of injected liquid along the injection track.

### Golgi staining

Animals were transcardially perfused with 100 ml of saline (0.9% NaCl in distilled water). Brains were immersed in Golgi–Cox solution (potassium dichromate (K_2_Cr_2_O_7_) 5%, mercuric chloride (HgCl_2_) 5%, potassium chromate (K_2_CrO_4_) 5%, and distilled water) for 6 days in the dark at 21 °C (using a glass scintillation vial). After this period of impregnation, the brains were left in 30% sucrose solution for at least 2 days. Gustatory cortex sections of 70 μm thickness were produced using a vibratome set to 5 mm/s, and amplitude of 5 mm. Slices were kept on microscope slides. For staining, slides were washed in ethanol 50–100%, ammonium hydroxyde 28–30%, sodium thiosulfate 1% in distilled water, distilled water, and xylene. Slides were dried for 1 h and then stored at 4 °C until further analysis.

### Dendritic protrusion analysis

Images were obtained using a Leica DMI6000 spinning disk microscope (PerkinElmer, Waltham, MA, USA; a gift from Fondazione Monzino) with a ×63 oil-immersion lens at a resolution of 1,024×1,024 pixels. Fiveto eight images were taken at depth intervals of 0.3 μm with ×1 zoom. The microscope acquisition parameters were kept identical for all images. Images were analyzed in a triple-blind manner using ImageJ (NIH). Dendritic protrusion density was measured by counting the number of dendritic protrusions on both primary and secondary dendrites and expressed as the number of dendritic protrusions per 10-μm dendritic length. The dendritic protrusion length was measured by manually drawing a vertical line from the protrusion’s tip to the point where it met the dendritic shaft and the head width was measured by drawing a line perpendicular to the length. Dendritic protrusions were measured in 10 neurons per animals. In the figure legends, *n* refers to the number of neurons quantified. Statistical significance was determined by Student’s *t* test.

### Statistical analysis

The results are expressed as means±s.e.m. For statistical analysis: Multiple comparison tests were applied with two-way ANOVA test with Scheffe *post hoc* analysis (Figures 1d, 2a, 2d, 4b). In addition, unpaired *t*-test for comparison between two groups was used (Figure 1a–d for differences between day 1 and day 3. Figure 3 with Welch correction; 5b,c. In cases where the results were not normally distributed, aparametric tests were used: Mann–Whitney (Figures 1c,d; 4a and 4b for differences between day 1 and day 3; Figure 4c,d) for comparing two groups, and Kruskal–Wallis for comparison between more than two groups (Figure 5a). The data were tested for normal distribution (Kolomogorov–Smirnov) and for equal variance (Levene Median). The majority of the data met the assumption except: Figure 1c,d for difference between day 1 and day 4; Figure 4a and 4b for difference between day 1 and day 3, Figure 3c,d; Figure 5a.

Sample size was calculated using a power calculator (http://biomath.info/power/) to ensure adequate power (>0.8) using estimations based on previous studies. No animals were excluded from analysis. No method of randomization was used for allocation of animals to experimental groups. Dendritic protrusion analysis was performed in a triple blind manner (experiment and staining, image acquisition, and image analysis were performed by three different co-authors), and all other experiments were performed in a non-blind manner.
